# TRPA1 and other TRP channels in migraine

**DOI:** 10.1186/1129-2377-14-71

**Published:** 2013-08-13

**Authors:** Silvia Benemei, Francesco De Cesaris, Camilla Fusi, Eleonora Rossi, Chiara Lupi, Pierangelo Geppetti

**Affiliations:** 1 Headache Center and Clinical Pharmacology Unit, Department of Health Sciences, Careggi University Hospital, University of Florence, viale Pieraccini 6, Florence 50139, Italy

**Keywords:** Migraine, Transient receptor potential ankyrin 1 (TRPA1), Transient receptor potential vanilloid 1 (TRPV1), Calcitonin gene-related peptide (CGRP), Neurogenic inflammation, ThermoTRP, Headache, Pain

## Abstract

Ever since their identification, interest in the role of transient receptor potential (TRP) channels in health and disease has steadily increased. Robust evidence has underlined the role of TRP channels expressed in a subset of primary sensory neurons of the trigeminal ganglion to promote, by neuronal excitation, nociceptive responses, allodynia and hyperalgesia. In particular, the TRP vanilloid 1 (TRPV1) and the TRP ankyrin 1 (TRPA1) are expressed in nociceptive neurons, which also express the sensory neuropeptides, tachykinins, and calcitonin gene-related peptide (CGRP), which mediate neurogenic inflammatory responses. Of interest, CGRP released from the trigeminovascular network of neurons is currently recognized as a main contributing mechanism of migraine attack. The ability of TRPA1 to sense and to be activated by an unprecedented series of exogenous and endogenous reactive molecules has now been extensively documented. Several of the TRPA1 activators are also known as triggers of migraine attack. Thus, TRP channels, and particularly TRPA1, may be proposed as novel pathways in migraine pathophysiology and as possible new targets for its treatment.

## Review

### TRP channels

The observation that the stimulation of a specific receptor, and the consequent, associated transient inward current [1,2] are necessary to vision in *Drosophila melanogaster* has been the primal evidence of the transient receptor potential (TRP) family of channels, which currently encompasses more than 50 different channels [3]. TRP channels represent a heterogeneous system oriented towards environment perception, and participating in sensing visual, gustatory, olfactive, auditive, mechanical, thermal, and osmotic stimuli. TRP channels consist of six transmembrane domains (S1-S6) with both the NH_2_ and COOH termini localized into the cytosol. The COOH region is highly conserved among TRPs, whereas the NH_2_ region usually contains different numbers of ankyrin repeats, the 33-residue motifs with a conserved backbone and variable residues that mediate protein-protein interactions [4]. The entry of cations through homo- or heterotetramers occurs *via* the pore formed by loops between the S5 and S6 domains. TRPs are commonly described as nonselective Ca^2+^-permeable channels, however, their Ca^2+^/Na^+^ permeability ratio may vary widely between different members of the TRP family [5].

TRP channel gating is operated by both the direct action on the channel of a plethora of exogenous and endogenous physicochemical stimuli, and changes in the intracellular machinery, including activation of G-protein coupled receptor (GPCR) or tyrosine kinase receptor [6]. In mammals, the TRP family consists of 28 proteins grouped into 6 subfamilies according to sequence identity, namely TRP canonical (TRPC), TRP vanilloid (TRPV), TRP melastatin (TRPM), TRP polycystin (TRPP), TRP mucolipin (TRPML), and TRP ankyrin (TRPA) [7,8]. The mammalian TRPC subfamily enlists 7 members (TRPC1-7), activated by the stimulation of GPCR and receptor tyrosine kinases [9], although TRPC1 seems to be directly activated by membrane stretch [10]. The TRPM has 8 members (TRPM1-8), and TRPM8 is activated by menthol and low temperatures (< 25°C). Of the three TRPML members, TRPML1 is widely expressed and has been described as an H^+^-sensor of endosomes/lysosomes, where it probably prevents overacidification [11]. The TRPP family can be subdivided into PKD1-like (TRPP1-like) and PKD2-like (TRPP2-like) proteins, which couple to act as a signaling complex at the plasma membrane plasma membrane [12]. Various diseases have been attributed to mutations of TRP channels. However, only a few TRP channelopathies, commonly known as “TRPpathies”, have been conclusively identified so far. TRPpathies include neurological disorders, renal diseases, and complex skeletal dysplasias [13].

### TRPA1 and other thermo-TRPs

Primary sensory neurons express different TRP channels, including four of the six members of the TRPV subfamily. TRPV1, TRPV2, TRPV3, and TRPV4 channels sense warm-hot temperatures and are chemosensors for a large series of naturally occurring and synthetic ligands. TRPV1, the receptor of the vanilloid compound capsaicin (which promoted the labeling of the group with the V), is responsive to high proton concentrations (pH 5–6) [14,15], anandamide [16] and various lipid derivatives [17]. Camphor and hypotonic solutions are non-selective activators of the TRPV3 and TRPV4, respectively [18,19]. The synthetic compound 4α-phorbol 12,13-didecanoate (4α-PDD), low pH, citrate, endocannabinoids, and arachidonic acid metabolites may also gate TRPV4 [20,21]. Activators of TRPV2 are not well identified, although the uricosuric agent probenecid can activate the channel [22]. Additional TRPs expressed in nociceptors are TRPA1 and TRPM8. Finally, there is also evidence that TRPM3, rather uniquely activated by pregnenolone sulfate, seems to be also expressed in primary sensory neurons [23].

A large NH2-terminal domain with 17 predicted ankyrin repeat domains characterizes TRPA1, the sole member of the TRPA subfamily. TRPA1, first cloned from human fetal lung fibroblasts, is widely expressed in mammals, where it has been found in hair cells, pancreas, heart, brain, keratinocytes [24], urinary bladder [25], prostate [26], arteries [27], enterochromaffin cells [28], odontoblasts and dental pulp [29,30], synovial fibroblasts [31], and epithelial and smooth muscle cells of the airways and lung [32]. A large amount of evidence shows that TRPA1 plays a key role in the detection of pungent or irritant compounds, including principles contained in different spicy foods, such as allyl isothiocyanate (mustard oil) in horseradish [33], allicin and diallyldisulfide in garlic [34], and cinnamaldehyde in cinnamon [35]. Gingerol (in ginger), eugenol (in cloves), methyl salicylate (in wintergreen), carvacrol (in oregano), thymol (in thyme and oregano) [36], are also able to gate TRPA1. In addition, environmental irritants and industry pollutants, such as acetaldehyde, formalin, hydrogen peroxide, hypochlorite, isocyanates, ozone, carbon dioxide, ultraviolet light, and acrolein (a highly reactive α,β-unsatured aldehyde present in tear gas, cigarette smoke, smoke from burning vegetation, and vehicle exhaust), have been recognized as TRPA1 activators [37-45]. The dispute regarding the role of TRPA1 as a sensor of mechanical stimuli and noxious cold (< 17°C) remains unresolved [36]. Electrophilic molecules have been found to activate TRPA1 *via* a unique mechanism, mediated by a Michael addition with specific cysteine and lysine residues identified in both the rat and human channel [46,47]. Finally, TRPA1-expressing neurons also express other TRP channels, in particular TRPV1, and, even more importantly, the sensory neuropeptides substance P (SP), neurokinin A (NKA), and calcitonin gene-related peptide (CGRP). SP/NKA and CGRP release from peripheral endings of nociceptors promoted by TRPV1 or TRPA1 activation produces a series of responses collectively described as neurogenic inflammation [48].

### TRPA1, pain and neurogenic inflammation

It is generally recognized that C and Aδ-fibre sensory neurons convey pain signals, while neurons with larger-size fibres mediate touch sensation. Thermo-TRPs, which under normal conditions are expressed in C and Aδ-fibre neurons, have been proposed to contribute to transmission and modulation of nociceptive signals. This conclusion originates from empirical observation that TRPV1 or TRPA1 agonists, derived from foods and spices, are able to cause, in a dose-dependent fashion, a range from appreciated hot feelings to unpleasant pain sensation, as in the case of capsaicin, the selective TRPV1 agonist contained in hot peppers, and piperine, another TRPV1 agonist present in black pepper, or allyl isothiocyanate, the TRPA1 agonist contained in mustard or wasabi [5,49]. Another finding that enlists thermo-TRPs as major pain controlling mechanisms is the clinical use of topical (cutaneous) capsaicin application that, by defunctionalizing sensory nerve terminals, alleviates several pain conditions, including post-herpetic neuralgias or pain associated with diabetic neuropathy [50].

Generation of deleted mice and, more importantly, identification and preclinical development of selective antagonists for thermo-TRPs, have greatly increased our knowledge of the role of these channels in the regulation of acute nociceptive responses and the development of allodynia and hyperalgesia. While TRPV1-deleted mice exhibit decreased hyperalgesia to elevated temperatures [51], TRPA1-deletion abrogated the two classical nociceptive phases to formalin [38]. However, gene deletion may not completely recapitulate the effects of channel blockade, as compensatory mechanism may counterbalance the function of the absent gene-protein. Thus, findings produced by using selective thermo-TRP antagonists may better unveil the role of these channels in models of pain diseases.

For detailed information on other thermo-TRPs, the reader is referred to previous review articles [5-8] whereas we will focus on the increasing evidence that supports the role of TRPA1 in models of both inflammatory and neuropathic pain. Mechanical hyperalgesia [52] and ongoing neuronal discharge [53,54] evoked by complete Freund’s adjuvant (CFA) and tumour necrosis factor-α (TNFα), and mechanical hyperalgesia induced by low doses of monosodium iodoacetate (MIA) [55] are inhibited by TRPA1 receptor antagonists in rodents. Convergent findings indicate that TRPA1 contributes to carrageenan-evoked inflammatory hyperalgesia [56,57]. Carrageenan administration, among a number of lipid derivatives, notably increases metabolites of 12-lipoxygenases, particularly hepoxilins A3 (HXA3) and HXB3, whose hyperalgesic/allodynic effect is abrogated by TRPA1 antagonism [58]. It has also been found that TRPA1 mediates ongoing nociception in chronic pancreatitis [59], and that both TRPV1 and TRPA1 initiate key pathways to transform acute into chronic inflammation and hyperalgesia in pancreatitis [60]. The clinical observation that an antioxidant afforded protection in patients with pancreatitis [61] indirectly supports the role of the oxidative stress sensor, TRPA1, in this condition.

The underlying mechanisms that from neural tissue injury produce the chronic allodynia and hyperalgesia typical of neuropathic pain are largely unknown. However, recent reports have pointed to the role of TRPA1 in different models of neuropathic pain. Several metabolic pathways in glycolysis or lipid peroxidation produce methylglyoxal (MG), which appears in the plasma in diabetic patients as hyperglycemia strongly enhances MG accumulation. MG has been recently described to react reversibly with cysteine residues, and probably due to this property stimulates TRPA1, thus representing a likely candidate metabolite to promote neuropathic pain in metabolic disorders [62]. Chemotherapeutic induced peripheral neuropathy (CIPN) is a scarcely understood and poorly treated condition, which, characterized by spontaneous pain, and mechanical and cold allodynia and hyperalgesia, causes significant discomfort, and often therapy discontinuation. CIPN may outlast the time period of chemotherapeutic drug administration for weeks or months [63].

Alteration of several ion channels has been advocated to explain this painful condition, but a univocal consensus has not emerged. Recently, in mouse models of CIPN produced by a single administration of oxaliplatin, paclitaxel or bortezomib, TRPA1 has been shown to play a major role in the development and maintenance of cold and mechanical [64-66]. In a therapeutic perspective, it is of relevance that treatment with a TRPA1 antagonist just before and shortly after (about 6 hours) the administration of bortezomib or oxaliplatin totally prevented the development and maintenance (for 10–15 days) of mechanical and cold hypersensitivity [66]. This finding suggests that TRPA1 is key in initiating CIPN and promoting the transition from an acute to a chronic condition. In addition, TRPA1 antagonists could represent novel therapeutic strategies of CIPN. In this context, it could be better understood that the unexpected attenuation by etodolac, a nonsteroidal anti-inflammatory drug of mechanical allodynia in a mouse model of neuropathic pain, might be due to its ability to inhibit TRPA1 [67]. In addition, to contribute to mechanical hyperalgesia, TRPA1 seems to be involved in the development of cold allodynia [68]. Cold allodynia was reduced in models of neuropathic (peripheral nerve injury) and inflammatory (CFA) pain [69,70] or in models of CIPN, which typically exhibit this type of hypersensitivity [64-66]. Interestingly, a familial episodic pain syndrome has been attributed to a gain of function mutation of TRPA1 TRPA1 [71].

**Figure 1:**
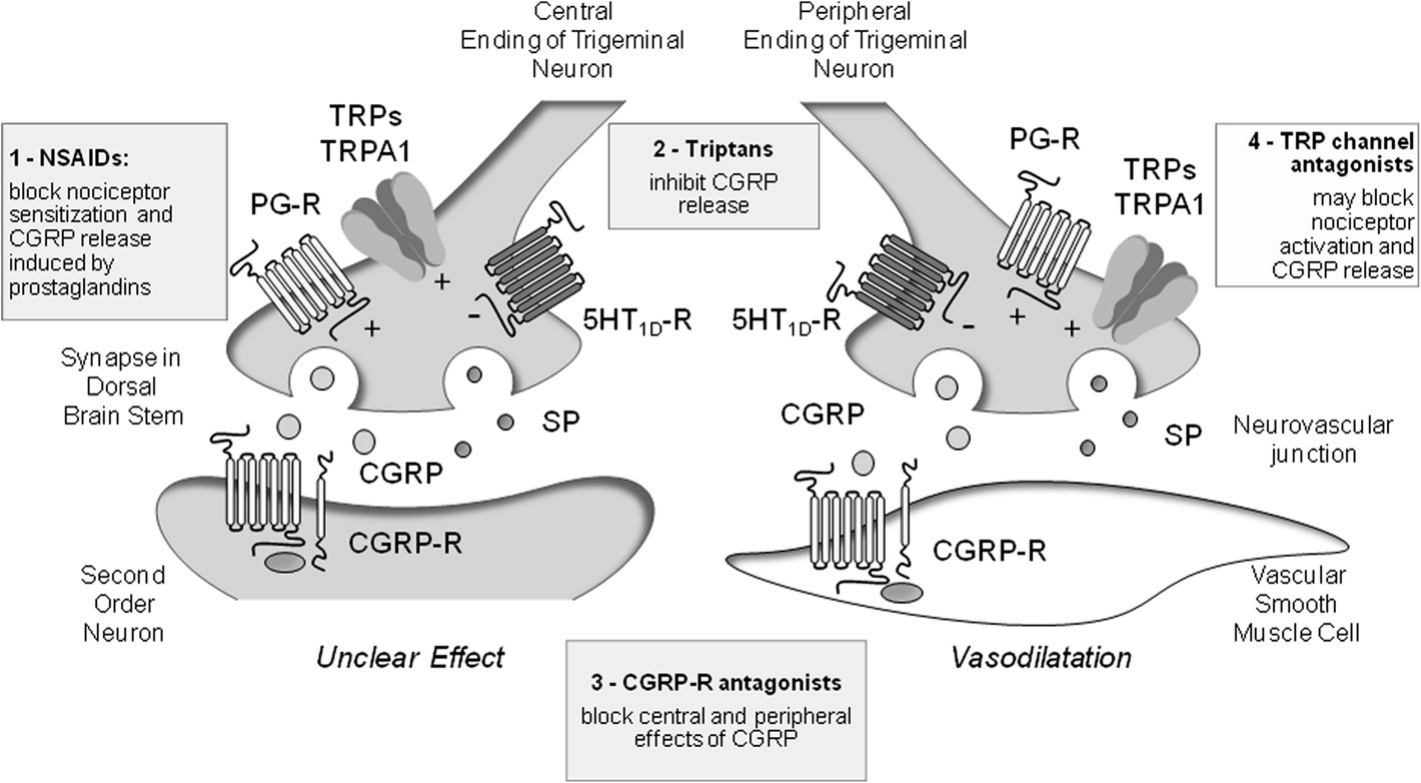
**Schematic representation of the probable mechanisms of the action of antimigraine remedies, either currently used, or proven effective in clinical trials (gray boxes) or of novel medicines (empty box), regarding their ability to modulate the release of calcitonin gene related peptide (CGRP) or the activation of its receptor (CGRP-R).** (1) Non steroidal antiinflammatory drugs (NSAIDs) block prostaglandin synthesis and the ensuing nociceptor sensitization and CGRP release evoked by prostaglandin receptor (PG-R) activation. (2) Triptans, by activating neuronal 5HT_1D_ receptors, inhibit CGRP release. (3) CGRP-R antagonists inhibit the action of CGRP on effector cells. (4) Antagonists of transient receptor potential channels (TRPs), including TRP ankyrin 1 (TRPA1), block the ability of a series of stimulants (for TRPA1, cigarette smoke, acrolein, nitric oxide, umbellulone, and others) to release CGRP. All medicines may act at both peripheral and central endings of trigeminal nociceptors. Receptor/channel activation may trigger/facilitate (+) or inhibit (−) CGRP release.

Coincidence between TRPA1 and neuropeptide expression has been suggested [72,73], although evidence for coexistence of isolectin B4-positive and non-peptidergic neurons with TRPA1 has also been reported [74,75]. Notwithstanding, exposure of tissues containing either peripheral or central sensory nerve terminals to TRPA1 agonists invariably results in a calcium-dependent release of SP/NKA and CGRP. Thus, TRPA1 stimulation has been proven to increase sensory neuropeptide release from oesophagus and urinary bladder, meninges, or dorsal spinal cord [76-78]. The outcome of such a release in peripheral tissues encompasses the series of responses commonly referred to as ‘neurogenic inflammation’ [48] (Figure 1). Although implication in transmission of nociceptive signals has been proposed, the pathophysiological outcome of the central release of sensory neuropeptides within the dorsal spinal cord or brain stem is less clear (Figure 1).

### TRPA1, TRPV1, and migraine

One of the first findings that, although indirectly, suggested the role of TRP channels in cluster headache and migraine was represented by the protective effect of the topical, desensitizing application of capsaicin to the patient nasal mucosa [79,80]. Capsaicin treatment couples the unique ability of the drug to first activate, and, subsequently, upon repeated administration, desensitize both the afferent pathway that from channel activation conveys nociceptive signals, and the ‘efferent’ function that results in sensory neuropeptide release [48]. Within the fifth cranial nerve, capsaicin desensitization causes the defunctionalization of peripheral and possibly central nerve endings, thus preventing SP/NKA-dependent plasma protein extravasation within the dura mater, CGRP-dependent dilatation of meningeal arterioles, and inhibition of afferent nociceptive impulses. Further support to the contribution of TRPV1 to migraine mechanism derived from the observation that ethanol, a known trigger of migraine attacks, activates CGRP release from sensory neurons, and promotes CGRP-dependent menin-geal vasodilatation by reducing the threshold temperature for channel activation, a phenomenon that eventually results in TRPV1 activation [81,82].

TRPV4 is stimulated by hypoosmotic stimuli that cause plasma membrane stretch and mechanical distension. Although this effect could, in principle, be implicated in the throbbing pain often described by migraine patients during their headache attacks, no evidence has yet been reported in support of TRPV4 in any model of head pain. In contrast, a series of observational findings has recently been obtained regarding a possible association between TRPA1 and migraine. In this respect, it is of interest that a number of compounds, recently identified as TRPA1 agonists, including cigarette smoke, ammonium chloride, formaldehyde, chlorine, garlic and others [34,38,41,78,83] are known triggers of migraine attacks in susceptible individuals [84-89].

Nitric oxide (NO) donor drugs, including nitroglycerine, are known inducers of migraine attacks and have been extensively used in migraine provocation studies in humans [90]. Although their pro-migraine action has been attributed to their intrinsic vasodilatatory action [91], the temporal mismatch between vasodilatation and the onset of migraine-like attacks argues against a close association between the two phenomena. In fact, at the time of the maximum vasodilatation, a mild to moderate headache develops both in migraineurs (more) and in healthy subjects (less), while delayed migraine-like attacks, present only in migraine patients, are observed only 5–6 hours after nitroglycerine administration [92,93]. Although not confirmed in vitro [94]. NO may release CGRP from trigemino-vascular neurons [95]. More recently, NO has been revealed to target TRPA1 by nitrosylation of channel cysteine residues [96], that seem to differ from those targeted by other reactive molecules [97], and this novel molecular mechanism could contribute to the nociceptive response evoked by NO [98]. It is possible that the S-nitrosylation process [99], produced by NO, contributes to channel sensitization to eventually (hours after the exposure to NO) amplify CGRP release by other agents, thus leading to exaggerated neurogenic inflammation and potentiation of pain responses.

The environmental pollution agent, acrolein, is produced by the combustion of organic material which causes its accidental inhalation, which, however, occurs also with cigarette smoking [100]. Several components of cigarette smoke, such as acrolein, crotonaldehyde [78], acetaldehyde [37] and nicotine [101], are TRPA1 agonists. Recently, acrolein application to the rat nasal mucosa has been shown to produce ipsilateral meningeal vasodilatation by a TRPA1- and CGRP-dependent mechanism [102], thus offering a mechanistic explanation for the association between the exposure to cigarette smoke and migraine attack appearance or worsening [103,104].

Herbalism has been instrumental for the development of pharmacology and also to a better understanding of pathophysiological mechanisms. These principles also apply to the migraine field. *Umbellularia californica* (California bay laurel) is also known as the ‘headache tree’ because of the ability of its scent to trigger headache attacks in susceptible individuals [105]. A case of cluster headache-like attacks preceded by cold sensations perceived in the ipsilateral nostril following inhalation of *Umbellularia californica* scent has recently been described in a cluster headache patient whose attacks had ceased 10 years before [106]. The irritant monoterpene ketone, umbellulone, one of the most abundant reactive molecules of *Umbellularia californica,* was found to activate the human recombinant and the constitutive rat/mouse TRPA1 in trigeminal ganglia (TG) neurons, and *via* this mechanism to produce nociceptive behaviour and the release of CGRP from TG or meningeal tissue in rats [77]. In addition, similar to acrolein, umbellulone application to the rat nasal mucosa evoked ipsilateral TRPA1- and CGRP-dependent meningeal vasodilatation [77].

Ligustilide, an electrophilic volatile dihydrophthalide of dietary and medicinal relevance, has been found to produce a moderate activation of TRPA1, but also to inhibit allyl isothiocyanate-evoked stimulation of TRPA1 [107]. This newly identified target of ligustilide offers a novel mechanistic explanation for the use of the compound in traditional medicine to treat pain diseases, including headaches. Finally, although several hypotheses have been advanced, the mechanism of the analgesic action of acetaminophen (paracetamol) in different pain conditions is far from clear. The reactive metabolite of acetaminophen, N-acetyl-p-benzo-quinoneimine (NAPQI), is able to activate the TRPA1 channel and thereby evoke a moderate and reversible neurogenic inflammatory response, which, in susceptible individuals, may contribute to emphasizing inflammation in peripheral tissues [76]. However, NAPQI may also be produced by cytochrome activity within the spinal cord, and NAPQI action at the spinal level results in channel desensitization [108]. Inhibition of central TRPA1 has been advocated as the mechanism, which may be responsible the hitherto unexplained analgesic and possibly antimigraine action of its parent molecule [108]. Importantly, this novel spinal mechanism could be of general relevance also for other TRPA1 agonists which share with NAPQI the ability of desensitizing the channel.

A host of endogenous inflammatory mediators possibly released during migraine attacks can activate and sensitize peripheral and central sensory neurons, including trigeminal neurons. Sensitization of first-order neurons is involved in the perception of headache throbbing pain [109], while sensitization of second‒order neurons contributes to cephalic allodynia and muscle tenderness [110,111]. Recently, it has been shown that innocuous brush and heat stimuli induce larger activation in the thalamus of patients who exhibit allodynia during mi-graine, as compared to pain‒free state, and that topical application of inflammatory molecules on the rat meninges sensitizes thalamic trigeminovascular neurons [112]. Each component of the nociceptive pathways could contribute differently to sensitization of the neural tissues. TRP channels could be sensitized by different compounds and *via* different mechanisms [5,36,66,81,113]. Although there is no specific information on the role of these channels to peripheral nociceptor sensitization or central sensitization in migraine, it is possible that TRP channels, and in particular TRPV1 and TRPA1, contribute to this key mechanism.

## Conclusions

The still largely unfinished puzzle of the migraine mechanism is being completed by novel unexpected pieces of information, which compose a clearer picture. The first, corner of the picture, known for decades, is that cyclooxygenase (namely cyclooxygenase 2) inhibition has a beneficial effect on migraine attack. The second corner, predicted by scientists and appreciated by clinicians and patients, is that targeting 5-HT1 receptors is also highly effective. The third corner is that a series of clinical trials show that CGRP antagonists afford a protection similar to that of triptans. To complete at least the frame, a fourth corner, which should reconcile the other three in an intelligible sequence of events, is needed. While prostaglandins sensitize nociceptors and eventually promote CGRP release, triptans inhibit such release, thus producing an indirect anti-migraine effect, and CGRP antagonists abrogate the final common pathway of migraine mechanism. The pathway, which, sensitized by prostaglandins and inhibited by serotonin receptor stimulation, results in trigeminal neuron activation and the pro-migraine release of CGRP could represent the fourth corner of the picture. Emerging information on TRP channels, and particularly TRPA1, which, targeted by migraine triggers, contribute, by activating the trigeminal CGRP-dependent pathway, to the genesis of pain and the accompanying symptoms of the attack, seems to be of paramount importance to solve what still remains the enigma of the migraine mechanism.

## Competing interests

The authors declare that they have no competing interests.

## Authors’ contribution

FDC, CF, ER, and CL searched for the literature. SB and PG wrote and revised the text. All authors read and approved the final manuscript.
